# PubMed Central archiving: a major milestone for a scholarly journal

**DOI:** 10.31138/mjr.31.1.3

**Published:** 2020-03-31

**Authors:** Armen Yuri Gasparyan, George D. Kitas

**Affiliations:** 1 Departments of Rheumatology and Research and Development, Dudley Group NHS Foundation Trust (Teaching Trust of the University of Birmingham, UK), Russells Hall Hospital, Dudley, West Midlands, United Kingdom,; 2 Arthritis Research UK Epidemiology Unit, University of Manchester, Manchester, United Kingdom

**Keywords:** PubMed, PubMed Central, periodicals as topic, publishing, quality control, open access, rheumatology

The *Mediterranean Journal of Rheumatology* (MJR) has recently achieved a major milestone for any “young” scholarly journal: it has been accepted for indexing by PubMed and archiving by PubMed Central (PMC). This is a major achievement that could not have been realized without the dedicated effort, hard work and support of our editorial team, reviewers, authors, and, of course, the vision and continuing support of the Greek Rheumatology Society and Professional Association of Rheumatologists. Journal editorial strategies require regular updates in view of the fast-changing global publishing trends, quality of journal submissions, and readership preferences. To successfully compete with numerous publication outlets in the field and better serve professional communities, journal editors should prioritize the quality and integrity of published contents that influence research, education, and practice. In the era of digitization and Open Access, registering journals with freely accessible scholarly platforms and permanently archiving on reliable repositories are becoming prerequisites of responsible and impactful publishing.^1^

In the field of biomedicine, PMC archiving is a major step towards global visibility and influence for journals and their contributors. Launched in 2000 by the US National Institutes of Health’s National Library of Medicine (NIH/NLM), PMC has become the most prestigious free repository of biomedical and life sciences journals that employ various open-access models. PMC as a reliable and freely accessible scholarly hub is gaining momentum in view of Plan S that will be enacted in 2021 to mandate immediate and free access to all publicly funded research.^2^ There are concerns that authors in developing countries and those lacking research funds are disadvantaged by Plan S and cut out of quality gold open-access journals.^3^ The latter, however, is largely compensated by the availability of platinum open-access journals, such as the MJR, where publishing and archiving charges are covered by professional societies, easing the authors’ and readers’ financial burden.^4^

Currently, PMC archives more than 2,200 fully participating journals ( https://www.ncbi.nlm.nih.gov/pmc/), which are selected after thorough scientific and technical evaluations. Being accepted for archiving is a mark of academic recognition and a credit, both requiring continuing efforts to fulfil the accepted obligations towards the global scholarly community. Journal editors and publishers should be aware that not all PMC-archived journals retain their status over time due to failing scientific standards. PMC articles are retrievable along with MEDLINE-indexed and NIH-funded items through the same PubMed platform that is arguably the main search point for the absolute majority of biomedical researchers, practitioners, and authors. The functionality of the PubMed platform allows recommending it for systematic syntheses of biomedical literature.^5^

Although the number of PMC articles constantly increases, not all publishers of biomedical journals are able to get their journals indexed by PubMed and archived by PMC. A large number of start-up open-access journals that fail to meet the NIH/NLM scientific and ethical standards are not successful with their applications.^6^ There are also remarkable precedents of discontinuing PMC archiving for journals with a long publishing history but failing scientific and editorial standards.^7^ The editors’ and publishers’ awareness of the PMC standards and other journals’ archiving history is helpful for current and future PMC applications.

To get archived and maintain their status, biomedical journals should regularly revise and upgrade their author instructions and publication ethics statements.^8^ For journal management teams that process submissions in research-intensive and pharma-sponsored fields, such as clinical rheumatology and immunology, it is mandatory to uphold their peer review and competing interest disclosure strategies. Overall, adhering to and enforcing updated editorial recommendations, such as those by the International Committee of Medical Journal Editors and the Council of Science Editors, can help pass the scientific evaluation.^9^ Choosing the most appropriate (liberal) copyrights and distribution licenses, assigning Digital Object Identifiers (DOI), formatting by Journal Article Tag Suite (JATS) eXtensible Markup Language (XML), and other essential adjustments for optimal open access can be supportive towards PMC archiving. Publishing articles in well-edited English is yet another requirement, which is particularly important for journals from non-Anglophone countries. Investing in substantive editing services may open up the PMC gates for a large number of such journals. Finally, validating article references and requesting relevant updates are also useful tactics given the fact that the PMC platform facilitates easy navigation to cross-linked references from PMC and MEDLINE-indexed articles.

Although PMC tracks citations, related metrics do not play a role for archiving. The citation tracking merely eases the online navigation within the PMC platform. At the same time, PMC archiving widens prospects of boosting article and journal traditional (citation) and alternative metrics. PubMed and PMC platforms are equipped with plug-ins for post-publication promotion through social media channels, such as Twitter and Facebook. Publicly available tweets and Facebook mentions expand the openness and implications of scholarly communications.^10^ Open-access journals with social media accounts are especially advantageous in that they can improve their immediate societal implications and build up on activities of their followers by regularly disseminating posts with open links to their articles.^11^ Journal editors concerned with post-publication promotion can benefit from PMC archiving by guiding their contributors how to actively share comments with open links and expand their social media networks.^12^

Importantly, the appearance of PMC articles on the PubMed platform reveals cross-links to semantically similar items, which may help the authors widen their collaboration prospects and guide the journal editors over the overlapping topics and redundancies.

Currently, the number of PMC journals listed by the NLM catalogue stands at 2,823 (https://www.ncbi.nlm.nih.gov/nlmcatalog/?term=journalspmcjournalspmc). Of these, 16 are in the field of rheumatology (https://www.ncbi.nlm.nih.gov/nlmcatalog/?term=journalspmc+and+rheum*). The list includes flagship gold open-access periodicals of the American and European rheumatology associations – *ACR Open Rheumatology* and *RMD Open*. The rest of the archived journals also predominantly employ a gold open access model, publishing articles with research funding and open-access fees. Only a few journals in the field, supported by regional societies, afford platinum open access – MJR has also achieved this with the continuing support of the Greek Rheumatology Society. These journals can be primarily targeted by researchers who lack research funds and archiving fees; the researchers can benefit from making their research and reviews discoverable and permanently archived.

Although PMC archiving is a major milestone for all successful rheumatology and allied journals, there remains a lot of work to do for journals, such as ours, that are still not indexed by major bibliographic databases, such as MEDLINE, Scopus and Science Citation Index Expanded. Merely opening access and archiving cannot secure journal quality and citability. As journal editors, we will continue to strive to get endorsements and supply of quality articles from related professional societies, to constantly improve the quality, transparency, and crediting of the peer review, and try and expand the geography of our authorship, so that we can claim that we deserve ‘whitelisting’ by concerned academic and research evaluation institutions.^13^
